# A rare case of inflammatory breast cancer with delayed auto-amputation in modern times

**DOI:** 10.1093/jscr/rjad159

**Published:** 2023-03-31

**Authors:** Hao Han Tan, Ian Y Goh, Geoffrey Muduioa

**Affiliations:** Department of General Surgery, Mater Hospital Brisbane, South Brisbane, QLD, Australia; Department of General Surgery, Mater Hospital Brisbane, South Brisbane, QLD, Australia; Faculty of Medicine, University of Queensland, Brisbane, QLD, Australia; Department of General Surgery, Mater Hospital Brisbane, South Brisbane, QLD, Australia

**Keywords:** inflammatory breast cancer, auto-amputation, salvage mastectomy, toilet mastectomy, global health, health inequity

## Abstract

Inflammatory breast cancer (IBC) is a rare but aggressive form of breast cancer, accounting for 0.5–2% of all diagnoses of invasive breast cancers. Yet, it is associated with very poor prognosis and outcomes, with documented 2- and 5-year survival rates around 84% and 40%, respectively, as compared to 90.6% of all breast cancers. Breast auto-amputation is also a rare complication of locally advanced breast cancer, associated with distressing symptoms for these patients. In this study, we report a 67-year-old female with a delayed diagnosis of IBC with a rare sequela of auto-amputation of the affected breast. The delay in diagnosis of >6 months led to a delay in the necessary treatment. She received neoadjuvant chemoradiotherapy and underwent a palliative right salvage mastectomy with level 2 axillary dissection.

## INTRODUCTION

Inflammatory breast cancer (IBC) is a rare but aggressive form of breast cancer, accounting for 0.5–2% of all diagnoses of invasive breast cancers [1, 2]. Patients with IBC typically present with rapidly progressing disease from self-diagnosed breast lumps. Most of them have lymph node involvement with around one-third having distant metastases [3]. Due to the underlying lymphoedema from the tumour emboli obstructing the dermal lymphatics, the skin over the breasts is usually erythematous, warm and thickened with a peau d’orange appearance.

Therefore, all patients should be properly worked up with a triple assessment, including history and physical examination, accompanied by medical imaging of ultrasonography and mammography, as well as a biopsy. Treatment of non-metastatic IBC involves multi-disciplinary discussion for a multimodal therapy, which includes neoadjuvant chemotherapy, mastectomy and post-mastectomy radiation. Even then, IBC still has a rather poor prognosis, with reported breast-cancer-associated deaths around 7–10% [1].

In this study, we report a 67-year-old female with a delayed diagnosis of IBC with a rare sequela of auto-amputation of the affected breast.

## CASE REPORT

A 67-year-old female was initially diagnosed in December 2012 with a mass in her right breast while residing in the Philippines. Due to personal reasons, she did not proceed with further investigations or surgical intervention. Her past medical history included hypertension, well-controlled type 2 diabetes and stable schizophrenia. There was no family history of breast cancer.

**Figure 1:**
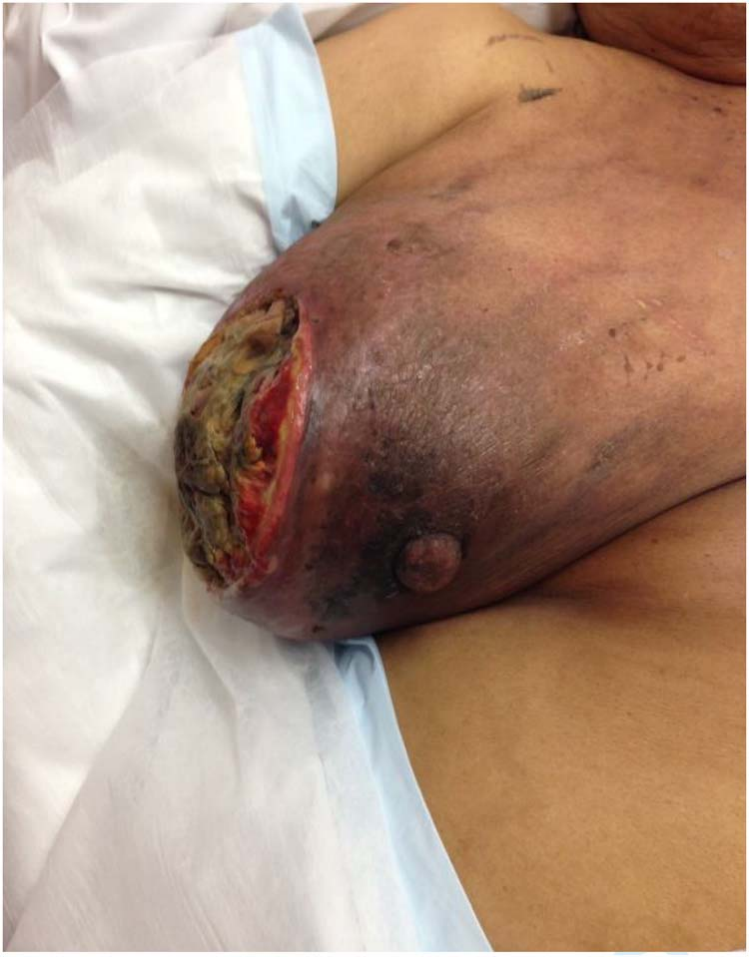
The fungating lesion on the right breast.

**Figure 2:**
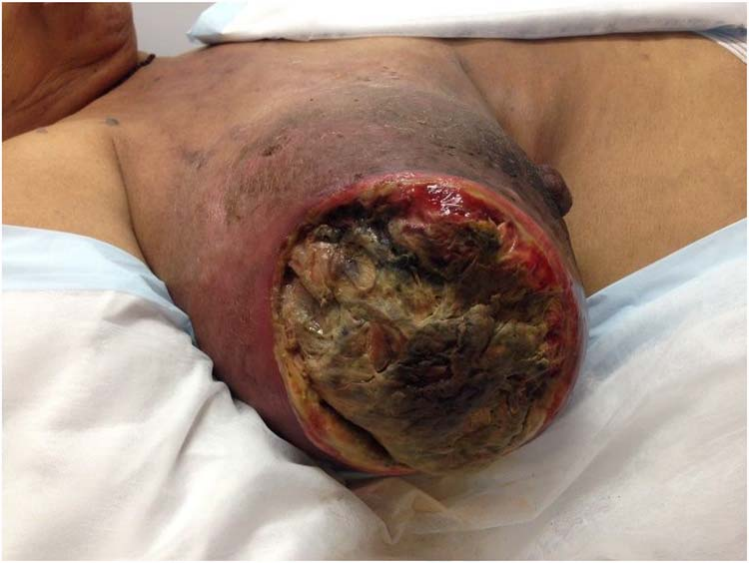
The fungating lesion on the right breast.

**Figure 3:**
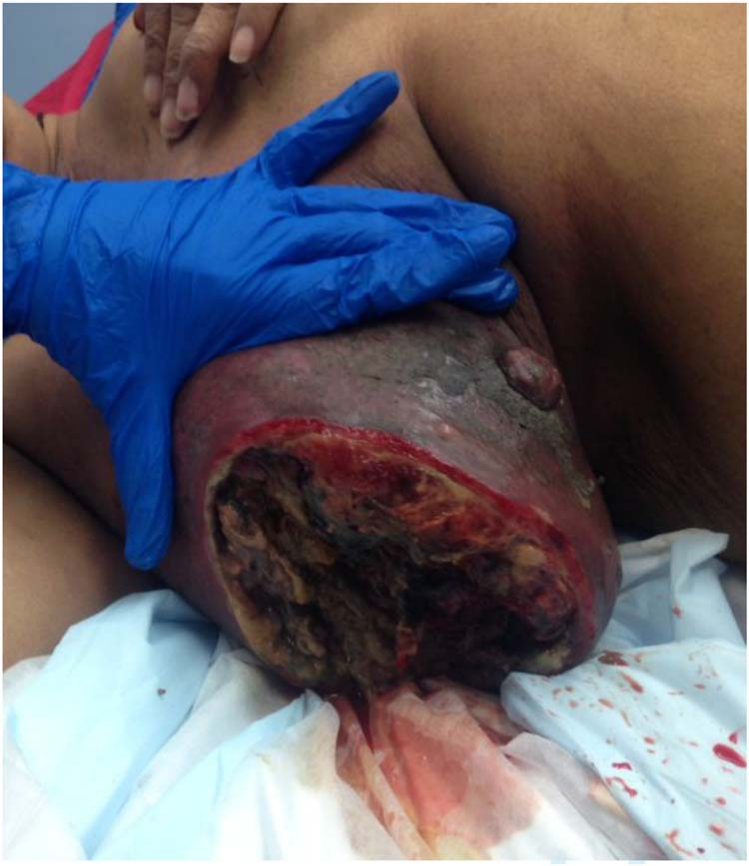
The auto-amputation of the same lesion after 2 days.

**Figure 4:**
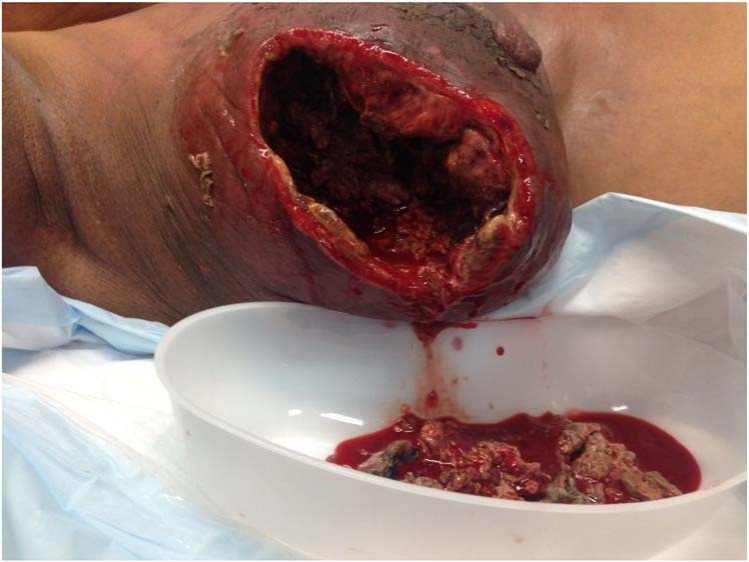
The resultant cavity.

She was then referred to a medical oncology outpatient clinic after returning to Australia in July 2013. Histological examination and immunochemistry revealed a triple negative, poorly differentiated right breast invasive ductal carcinoma, with no evidence of metastatic disease on both computed tomography and bone scans. She was commenced on neoadjuvant chemotherapy (Adriamycin, Cyclophosphamide and Docetaxel). She also received neoadjuvant radiotherapy in her treatment. Despite treatment, her tumour continued to progress and doubled in size in the span of 3 months. During her second cycle of neoadjuvant treatment, her progress was complicated by right breast cellulitis that eventually progressed into necrosis despite intravenous antibiotics. It eventually developed into a large fungating lesion at the lateral aspect of her nipple, which led to an acute complete auto-amputation of the breast tissue, leaving a large necrotic cavity (Figs 1–4).

**Figure 5:**
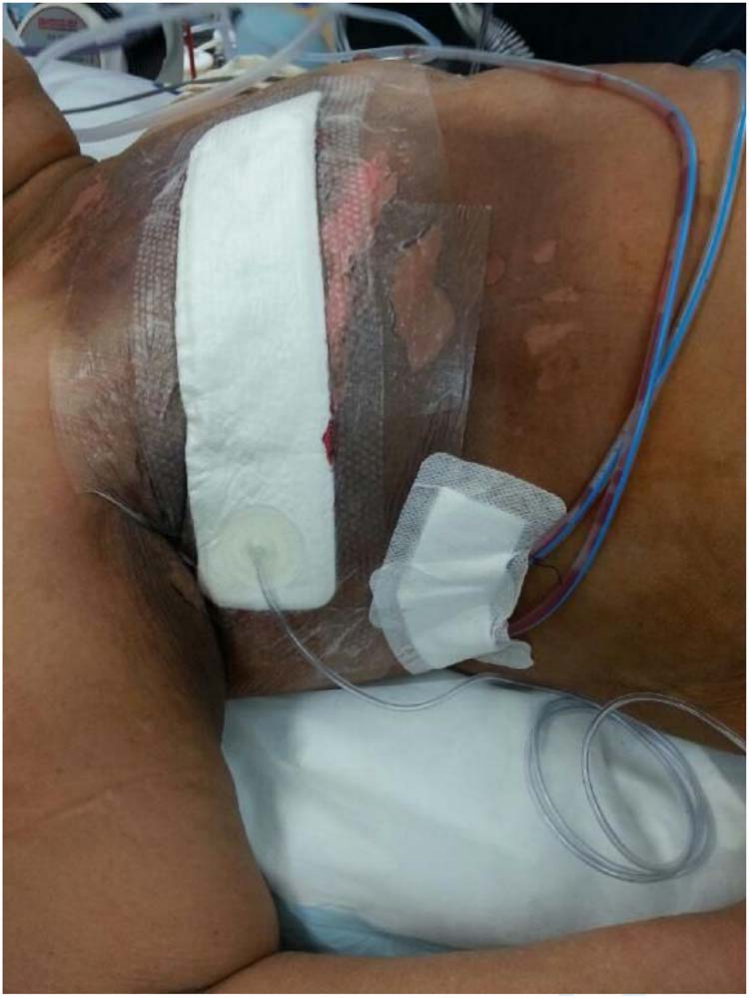
The figure was taken after the surgery (right salvage mastectomy with level 2 axillary dissection) with vacuum dressing and drains *in situ*.

The general surgical team was consulted, and she later progressed to have a right salvage mastectomy with level 2 axillary dissection during the same admission (palliative nature rather than curative) (Fig. 5). Intraoperatively it was noted that the locally advanced tumour was involving the pectoralis major muscle with significant neovascularisation, resulting in almost 800 ml of blood loss requiring multiple blood transfusions. A total of 935 mg of breast tissue was excised. The histology was reported as stage pT4bN0 (a 75-mm Grade 3 metaplastic carcinoma with no lymphovascular space invasion, triple-negative receptors, Grade 3 histologic response to chemotherapy between 30% and 90% reduction in tumour cells and all 12 lymph nodes were negative).

Post-operatively, her recovery was complicated by wound dehiscence, poor wound healing and secondary wound infection. This required a surgical debridement in theatre with multiple courses of antibiotics. The wound eventually healed after a prolonged duration of vacuum dressings.

Her case was discussed at the breast multi-disciplinary teams meeting with the consensus being for further adjuvant chemotherapy. She remained disease-free for 6 months post-mastectomy but passed away 3 months later from other health-related issues.

## DISCUSSION

IBC is a rare form of breast cancer with poor prognosis and outcomes. Even with treatment, the 2-year survival rate is around 84% compared to 91% in non-IBC, with a 5-year survival rate of around 40% compared to 90.6% of all breast cancers [4, 5].

Due to health inequity, breast cancer diagnosis in developing countries (DCs) represents around half of the incidence but accounts for more than 60% of all deaths [6]. Breast cancer continues to remain the leading cause of death in females in DCs. Although medical knowledge and technology have advanced drastically in developed countries, the same might not apply to the rest of the world. In this case, her diagnosis was actually delayed by more than 6 months, resulting in the delay in necessary treatment. This was also likely compounded by poor health literacy and access to healthcare. Extra level of care and vigilance should be warranted for immigrants presenting with such symptoms in Australia to ensure that they are not lost to follow-up. Earlier diagnosis and treatment might have resulted in a more optimistic outcome.

Breast auto-amputation is a rare occurrence and has only been reported in the literature as a series of case reports. Most of them were from DCs such as South Africa and Nigeria, with the most recent case report being from Turkey [7]. As far as our knowledge goes, this is the first-ever case report of breast auto-amputation in Australia. It is difficult to know the full causative pathophysiology of the auto-amputation in this patient context. The process might have been triggered off by necrosis of the skin with superimposed tumour necrosis. Following that, the extensive destruction of the deeper structures leads to the eventual detachment of the breast tissue from the thoracic wall [7].

In this case, radiotherapy and superimposed infection could have predisposed the patient to the auto-amputation phenomenon.

Finally, there are various techniques described for mastectomies, including simple, skin-sparing, palliative and radical. Palliative mastectomy, also known as toilet mastectomy, remains a key adjunct to palliative care in breast cancer patients. The aim is to control local complications from metastatic and locally advanced breast cancer that are distressing for the patients, such as bleeding, pain, slough and secondary infections [8]. The patient was able to achieve satisfactory relief from her complications with an improved quality of life as a result.

## CONCLUSION

IBC is a rare form of breast cancer with a poorer prognosis and outcomes. Breast auto-amputation is a rare complication of locally advanced breast cancer, associated with distressing symptoms for these patients. Unfortunately, the incidence of these conditions is much higher in DCs due to the innate nature of health inequity with lack of screening in these countries.

## CONFLICT OF INTEREST STATEMENT

The authors declare that there is no conflict of interest.

## FUNDING

The authors received no financial support for the publication of this article.

## DATA AVAILABILITY STATEMENT

All data are incorporated into the article and its online supplementary material.

## References

[1] Hance KW, Anderson WF, Devesa SS, Young HA, Levine PH. Trends in inflammatory breast carcinoma incidence and survival: the surveillance, epidemiology, and end results program at the National Cancer Institute. J Natl Cancer Inst 2005;97:966–75. 15998949 10.1093/jnci/dji172 PMC2844937

[2] Anderson WF, Schairer C, Chen BE, Hance KW, Levine PH. Epidemiology of inflammatory breast cancer (IBC). Breast Dis 2005;22:9–23. 16735783 10.3233/bd-2006-22103 PMC2852616

[3] Walshe JM, Swain SM. Clinical aspects of inflammatory breast cancer. Breast Dis 2005;22:35–44. 16735785 10.3233/bd-2006-22105

[4] Dawood S, Ueno NT, Valero V, Woodward WA, Buchholz TA, Hortobagyi GN. et al. Differences in survival among women with stage III inflammatory and noninflammatory locally advanced breast cancer appear early: a large population-based study. Cancer 2011; 117:1819–26. 21509759 10.1002/cncr.25682

[5] American Cancer Society . Inflammatory Breast Cancer [Internet]. American Cancer Society; 2021 (Updated 1 March 2022; cited 31 January 2023). Available from: https://www.cancer.org/cancer/breast-cancer/about/types-of-breast-cancer/inflammatory-breast-cancer.html.

[6] Torre LA, Bray F, Siegel RL, Ferlay J, Lortet-Tieulent J, Jemal A. Global cancer statistics, 2012. CA Cancer J Clin. NW, Atlanta; 2015;65:87–108. 25651787 10.3322/caac.21262

[7] Ergül N, Kadıoğlu H, Yıldız S, Başkaya Yücel S, Aydın M. Autoamputation of breast caused by invasive ductal carcinoma. Rev Esp Med Nucl Imagen Mol 2013;32:199–200. 22877570 10.1016/j.remn.2012.06.005

[8] Radziszewski M, Choromańska E, Nowak A, Kowalewski R, Radziszewski J. Toilet mastectomy as a possible and recommended solution to improve the quality of life of advanced breast cancer patients – case series. Medycyna Paliatywna/Palliat Med 2021;13:97–100.

